# Second-Generation Sequencing with Deep Reinforcement Learning for Lung Infection Detection

**DOI:** 10.1155/2020/3264801

**Published:** 2020-02-22

**Authors:** Zhuo Liu, Gerui Zhang, Zhao Jingyuan, Liyan Yu, Junxiu Sheng, Na Zhang, Hong Yuan

**Affiliations:** The First Affiliated Hospital of Dalian Medical University, Dalian 116011, China

## Abstract

Recently, deep reinforcement learning, associated with medical big data generated and collected from medical Internet of Things, is prospective for computer-aided diagnosis and therapy. In this paper, we focus on the application value of the second-generation sequencing technology in the diagnosis and treatment of pulmonary infectious diseases with the aid of the deep reinforcement learning. Specifically, the rapid, comprehensive, and accurate identification of pathogens is a prerequisite for clinicians to choose timely and targeted treatment. Thus, in this work, we present representative deep reinforcement learning methods that are potential to identify pathogens for lung infection treatment. After that, current status of pathogenic diagnosis of pulmonary infectious diseases and their main characteristics are summarized. Furthermore, we analyze the common types of second-generation sequencing technology, which can be used to diagnose lung infection as well. Finally, we point out the challenges and possible future research directions in integrating deep reinforcement learning with second-generation sequencing technology to diagnose and treat lung infection, which is prospective to accelerate the evolution of smart healthcare with medical Internet of Things and big data.

## 1. Introduction

Nowadays, smart healthcare has appeared to be an interdisciplinary subject by integrating mixed computing techniques into the health administration [1, 2]. The primary purpose of smart healthcare is to offer pervasive and personalized medical services and health protection to people. Computer-aided diagnosis and decision making of this personalized treatment plan is one of the current developments in precision medicine [3, 4]. Smart healthcare aims to provide intelligent comprehensive differentiation and prescription recommendation for the diagnosis and treatment of diseases by applying artificial intelligence technology and cloud computing to the practice of clinical medicine. It has been greatly developed through the applications of artificial intelligence, cloud computing, big data analysis, and Internet of Things (IoT), and has been applied to many medical fields such as intelligent Chinese medicine and intelligent testing. Medical big data is to integrate the IoT system into medicine and to integrate and classify the collected medical data information by creating the medical Internet of Things [5, 6]. The deep learning model and the deep reinforcement learning model are the most commonly used artificial intelligence models, which can be trained and simulated by providing a large number of training examples through medical big data. The computer aids of modern medicine and traditional Chinese medicine have matured. Thus, there are many well-trained deep learning models for clinical medicine.

Pulmonary infectious diseases are common respiratory diseases, whose clinical manifestations include cough, fever, and chills. However, pathogens of lung infections are complex, and it is difficult to carry out biological cultivation and identification. Particularly, complex lung infections have various clinical manifestations: the mortality rate is high and the treatment is difficult, the traditional pathogen detection methods have low positive rate; it is a long time-consuming and complicated operation, and it is difficult to meet the requirements of diagnosis and treatment of complex infectious diseases. Classical pathogen detection methods include bacterial (fungal) culture, microscopy and antibody testing, and PCR-based pathogen-specific nucleic acid detection. These methods have made great progress in the diagnosis of pulmonary infectious diseases, but they have low sensitivity and poor timeliness; pathogen identification information and drug resistance information are not comprehensive, so it is impossible to identify unknown and rare pathogenic microorganisms. Therefore, clinical diagnosis of pulmonary infectious diseases is very difficult.

Second-generation sequencing (SGS), also known as next-generation sequencing technology and high-throughput sequencing, can simultaneously sequence billions of DNA molecules in parallel [7]. It is a group of sequencing with high throughput, low cost, short time, and automated sequencing technologies [8–10]. However, poor specificity is a major problem that restricts the clinical applications of SGS. Nonpathogenic pathogens, unrelated pathogens, and ambiguous pathogens are often seen in SGS reports. In order to clarify the diagnosis, the pathogen information detected by SGS needs to be verified and interpreted using more advanced methods [11, 12].

In this paper, we explore deep reinforcement learning for computer-aided diagnosis and treatment of complex pulmonary infectious diseases. We present several representative deep reinforcement learning models for the diagnosis and treatment of potential lung infections first, discuss the applications of the deep reinforcement learning model in the diagnosis of second-generation genetic testing for pulmonary infection, and summarize current status of pathogenic diagnosis of pulmonary infectious diseases and their main characteristics, and then we analyze the results of second-generation genetic testing and the main features of each type in common lung infections. Finally, we point out the open challenges and possible future research directions for intensive studies of second-generation genetic testing integrated with deep reinforcement learning in lung infections, which is expected to promote the development of intelligent healthcare and medical Internet of Things.

In the reminder of this paper, Section 2 introduces the deep reinforcement learning approaches that can be used for second-generation sequencing for lung diseases detection and treatment.  Section 3 reviews the current status of pathogenic diagnosis of pulmonary infectious diseases and applications of SGS in detection of pulmonary pathogen infection are discussed in Section 4.  Section 5 gives the challenges and possible future research directions for intensive studies of second-generation genetic testing integrated with deep reinforcement learning in lung infections. Finally, Section 6 concludes the paper.

## 2. Deep Reinforcement Learning

**Figure 1:**
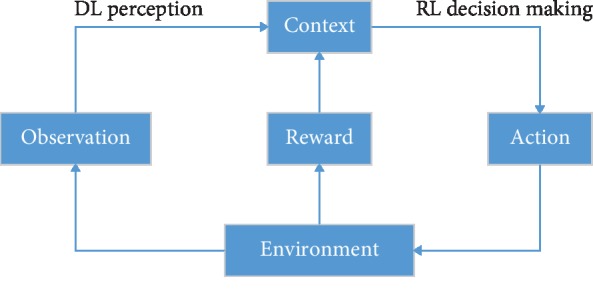
The framework of deep reinforcement learning.

Deep reinforcement learning (DRL) is a new research hotspot in the field of artificial intelligence. It combines the perception of deep learning with the decision-making ability of reinforcement learning in a common form and enables direct control from raw input to output through end-to-end learning. With the rapid development of human society, in more and more complex real-world task tasks, deep learning (DL) is needed to automatically learn the abstract representation of large-scale input data and to use this representation as a basis for self-incentive reinforcement learning (RL) to optimize problem-solving strategies. DRL is an end-to-end sensing and control system with strong versatility. The learning process can be described as follows: (1) At each moment, the agent interacts with the environment to obtain a high-dimensional observation and uses the DL method to perceive the observation to obtain a specific state feature representation. (2) The value function of each action is evaluated based on the expected return, and the current state is mapped to the corresponding action through a certain strategy. (3) The environment reacts to this action and gets the next observation. By continuously cycling the above processes, the optimal strategy for achieving the goal can be finally obtained [13]. The framework of DRL is shown in Figure 1.

At present, DRL technology has been widely used in games, parameter optimization, machine vision, and other fields. Its application is considered as an important way to move toward general artificial intelligence [14, 15]. This paper explores the application of DRL in second-generation sequencing for lung infection detection.

### 2.1. Basic Concepts

#### 2.1.1. Deep Learning

The concept of DL stems from artificial neural networks. The DL model is usually composed of multiple layers of nonlinear arithmetic units. It uses the output of the lower layer as the input of the higher layer; in this way, it automatically learns the abstract feature representation from the large amount of training data to discover the distributed characteristics of the data. Compared with shallow networks, multi-hidden-layer network models have better feature representation capabilities. It first uses the unsupervised learning to conduct greedy pretraining on layer by layer and then supervises the whole network with supervised learning. This pretraining method provides ideal initial parameters for deep neural networks and reduces the optimization difficulty of deep neural networks [16, 17]. Typical DL models include Stacked Autoencoder (SAE), Restricted Boltzmann Machine (RBM), Deep Belief Network (DBN), and Recurrent Neural Network (RNN). With the growth of training data and the improvement of computing power, Convolutional Neural Network (CNN) has been widely used in various fields.

#### 2.1.2. Reinforcement Learning

Reinforcement learning (RL) is a kind of learning that maps from environmental state to action. The goal is to get the agent to get the maximum cumulative reward in the process of interaction with the environment [18]. The Markov decision process can be used to model the RL problem, which is usually defined as (*S*, *A*, *ρ*, *f*), where

1. *S* is a collection of all environmental states. And *s*_*t*_ ∈ *S* stands for the state of agent at time *t*.
2. *A* is a collection of execution actions for agent. And *a*_*t*_ ∈ *A* stands for the action that agent takes at time *t*.
3. *ρ* : *S* × *A*⟶*R* is the reward function. And *r*_*t*_ ~ *ρ*(*s*_*t*_, *a*_*t*_) stands for the immediate reward value of agent at state *s*_*t*_ when executing action *a*_*t*_.
4. *f* : *S* × *A* × *S*⟶[0,1] is the state transition probability distribution function. And *s*_*t*+1_ ~ *f*(*s*_*t*_, *a*_*t*_) stands for the probability of agent transforming from state *s*_*t*_ to *s*_*t*+1_ when executing action *a*_*t*_.

In RL, strategy *π* : *S* × *A* is a map from state space to action space, which indicated that the agent selects action *a*_*t*_ in state *s*_*t*_, performs the action, and transforms to the next state *s*_*t*+1_ with probability *f*(*s*_*t*_, *a*_*t*_), while accepting rewards *r*_*t*_ from environmental feedback. Assuming that the immediate reward for each time step in the future must be multiplied by a discount factor *γ*, then from the time *t* to the end of the time *T*, the sum of the rewards is defined as

(1) $$\begin{array}{c} R_{t}=\sum_{i=t}^{T}\gamma^{i-t}r_{i}, \end{array}$$

in which *γ* ∈ [0,1] is used to weigh the impact of future rewards on cumulative rewards.

State action value function *Q*^*π*^(*s*, *a*) refers to the action *a* in the current state *s* and always follows the strategy *π* to the end of the plot, in which the cumulative return obtained by the agent is expressed as

(2) $$\begin{array}{c} Q^{\pi }\left(s,a\right)=Ε\left[R_{t}\left|s_{t}\right.=s,a_{t}=a,\pi \right]. \end{array}$$

For all state action pairs, if the return of a strategy *π*^*∗*^ is greater than or equal to the expected return of all other strategies, then the strategy *π*^*∗*^ is called the optimal strategy. There may be more than one optimal strategy, but they share the same state action value function.

(3) $$\begin{array}{c} Q^{\pi }\left(s,a\right)=\underset{\pi }{\max}Ε\left[R_{t}\left|s_{t}\right.=s,a_{t}=a,\pi \right]. \end{array}$$

It is called the optimal state action value function, and the optimal state motion value function follows the Bellman optimal equation; namely,

(4) $$\begin{array}{c} Q^{\pi }\left(s,a\right)={Ε}_{s^{\prime }:S}\left[r+\gamma \underset{a^{\prime }}{\max}Q\left(s^{\prime },a^{\prime }\right)\left|s,a\right.\right]. \end{array}$$

In the traditional RL, the *Q*-value function is generally obtained by iterative Bellman equation:

(5) $$\begin{array}{c} Q_{i+1}\left(s,a\right)={Ε}_{s^{\prime }:S}\left[r+\gamma \underset{a^{\prime }}{\max}Q_{i}\left(s^{\prime },a^{\prime }\right)\left|s,a\right.\right]. \end{array}$$

Herein, when *i*⟶*∞*, *π*^*∗*^. By continuously iterating, the state action value function will finally converge, and the optimal strategy $\pi^{*}=\underset{a\in A}{\arg\max}Q^{*}\left(s,a\right)$ will be obtained. However, for practical problems, it is obviously not feasible to solve the optimal strategy by iterative updating (5), because in the large state space, the method of solving the *Q*-value function with the iterative Bellman equation is too expensive. To tackle it, in the RL algorithm, a linear function approximator is usually used to approximate the state action value function, *Q*(*s*, *a*|*θ*) ≈ *Q*^*∗*^(*s*, *a*). Besides, nonlinear function approximators such as deep neural networks can also be used to approximate the value function or strategy. Therefore, DRL has attracted extensive attention in recent years. In the next subsection, we will discuss some deep reinforcement learning techniques that are potential for second-generation sequencing in lung infection detection and treatment.

### 2.2. DRL Techniques

In this section, we first describe three main types of deep reinforcement learning methods, including deep reinforcement learning based on value function, deep reinforcement learning based on strategy gradient, and deep reinforcement learning based on search and supervision. Afterwards, some potential deep reinforcement learning directions in SGS applications are summarized, such as hierarchical deep reinforcement learning, multitask deep reinforcement learning, multiagent deep reinforcement learning, deep reinforcement learning based on memory and reasoning, and so on.

#### 2.2.1. DRL Based on Value Function

**Figure 2:**
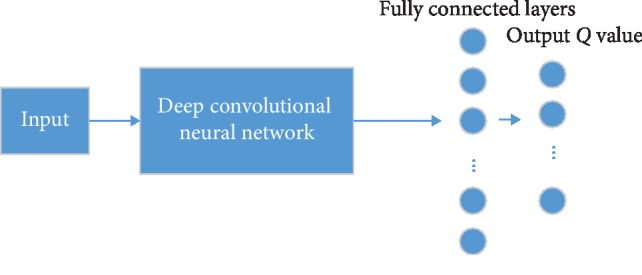
Architecture of DQN.

Mnih et al. [19] combined the convolutional neural network with the *Q* learning algorithm in the traditional RL and proposed the Deep Q-Network (DQN) model. This model is used to process visual perception-based control tasks and is a groundbreaking work in the field of DRL. The input of the DQN model is the four preprocessed images closest to the current time. The input undergoes a nonlinear transformation of 3 convolutional layers and 2 fully connected layers and finally produces a Q value for each action in the output layer.  Figure 2 shows the architecture of DQN.

In order to alleviate the instability problem in the nonlinear network representation value function, DQN mainly made three improvements to the traditional *Q* learning algorithm. (1) DQN uses the experience replay mechanism during the training process to tackle the obtained transferred samples online. (2) In addition to using the deep convolutional network to approximate the current value function, DQN uses another network to generate the target *Q* value. (3) DQN reduces the bonus value and error term to a limited interval, which ensures that the Q value and the gradient value are within a reasonable range, which improves the stability of the algorithm. Inspired by the DQN, many variants are proposed, such as deep dual *Q* network, deep *Q* network based on superior learning, deep *Q* network based on priority sampling, deep cycle *Q* network, and so on.

#### 2.2.2. DRL Based on Strategy Gradient

Strategy gradient is a commonly used strategy optimization method, which updates the strategy parameters by continuously calculating the gradient of the strategy expectation total reward for the strategy parameters and finally converges to the optimal strategy. Therefore, when solving the DRL problem, a deep neural network with parameter *θ* can be used to parameterize the representation strategy, and the strategy gradient method is used to optimize the strategy. It is worth noting that when solving DRL problems, the first choice is to adopt a strategy-gradient-based algorithm. The reason is that it can directly optimize the expected total reward of the strategy and search for the optimal strategy directly in the strategic space in an end-to-end manner, eliminating the cumbersome intermediate links. Therefore, compared with DQN and its improved model, the DRL method based on strategy gradient is more applicable and the effect of strategy optimization is better.

Typical strategy-gradient-based DRL methods include deep strategy gradient based on actor critic, asynchronous dominant actor critic algorithm, and so on [20].

#### 2.2.3. DRL Based on Search and Supervision

In addition to value-based DRL and strategy-gradient-based DRL, the process of strategy search can be promoted by adding additional manual supervision, which is the core idea of DRL based on search and supervision. Monte Carlo Tree Search (MCTS) [21], as a classic heuristic strategy search method, is widely used in action planning in game problems. Therefore, in the DRL method based on search and supervision, strategy search is generally done through MCTS. For example, the AlphaGo algorithm combines deep neural networks with MCTS to achieve remarkable results. Its main idea has two points: (1) using MCTS to approximate the value function of each state; (2) using the CNN based on value function to evaluate the current layout and walk of the board. AlphaGo's complete learning system consists of the following components:

1. *Strategy Network*. It is divided into a strategy network for supervised learning and a strategy network for RL. The role of the strategy network is to predict and sample the next move based on the current situation.
2. *Rollout Strategy*. The goal is also to predict the next step, but the speed of prediction is 1000 times that of the strategy network.
3. *Value Network*. According to the current situation, the winning probability of both sides is estimated.
4. *MCTS*. It integrates the strategy network, the rollout strategy, and the value network into the process of strategy search to form a complete system.

DRL based on search and supervision has achieved promising results in the game areas, which has prompted more and more researchers to transfer it to others.

#### 2.2.4. Potential DRL in SGS

In addition to the above DRL methods, some outstanding methods have be proposed in recent years. In this subsection, we give a brief review of the potential DRL in SGS, which may be used for detecting of pulmonary pathogen infection.

**Figure 3:**
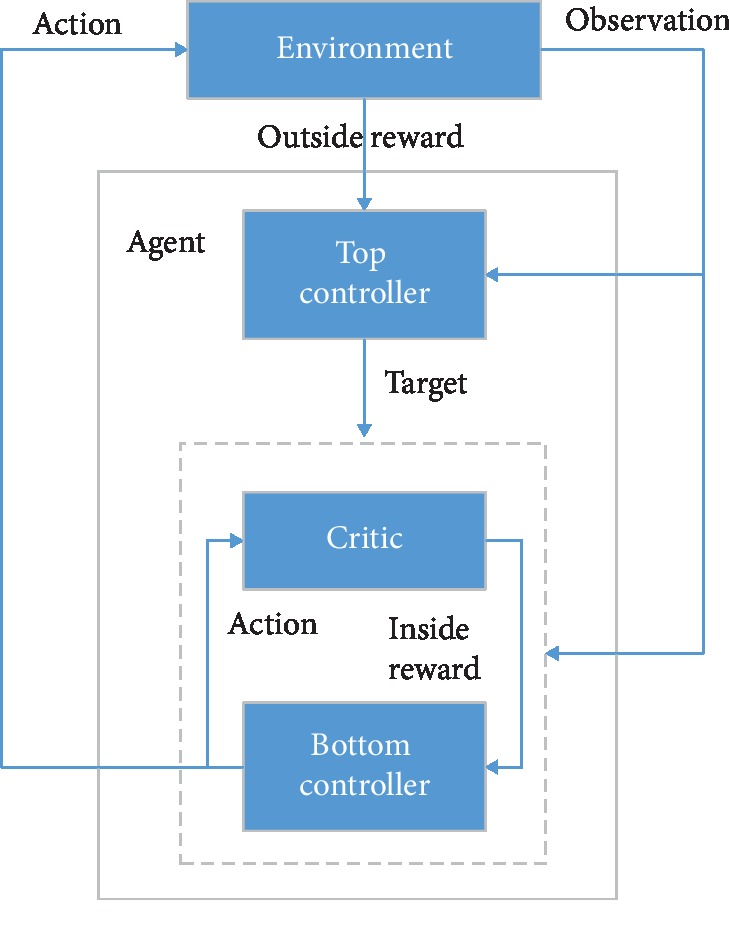
Architecture of hierarchical DQN.

*(1) Hierarchical Deep Reinforcement Learning*. In some complex DRL tasks, the strategy is optimized directly to the final goal, which is inefficient. Therefore, Hierarchical Reinforcement Learning (HRL) can be used to decompose the final goal into multiple subtasks to learn the hierarchical strategy and form a valid global strategy by combining multiple subtask strategies [22].  Figure 3 gives the structure of the hierarchical DQN.

Typical HRL methods include spatiotemporal abstraction and intrinsic-motivation-based methods, internal-option-based methods, and deep follow-up reinforcement learning. All the ideas can be used with the complex processes of SGS for detection of pulmonary pathogen infection.

*(2) Multitask Transfer Deep Reinforcement Learning*. In the traditional DRL method, the agent after completion of each training can only solve a single task. However, in some complex real-world scenarios, the agent needs to be able to handle multiple tasks at the same time. At this time, multitask learning and transfer learning are extremely important. In the RL field, Wilson et al. [23] used a hierarchical hybrid Bayesian model to provide prior knowledge of new tasks, enabling agents to better adapt to new task scenarios. For partially observable random multitasking scenarios, Li et al. [24] developed a regionalized policy representation to describe the behaviour of agents in different task scenarios. The method used the clustering properties contained in the Dirichlet process to share training scenarios between similar tasks and to pass valuable information between different tasks. Compared with the single-task learning mode, the multitask RL method has achieved more outstanding performance in both lattice world navigation and multitarget classification tasks. Taylor and Stone [25] proposed a way to transfer value functions between different tasks. Fernfindez Vdoso [26] used a mapping that reflects the relationship between the agent's current and past state action pairs, enabling the previously learned strategies to be transferred to new tasks in a timely manner. Wang et al. [27] concluded that the transfer learning in RL falls into two broad categories: behavioural transfer and knowledge transfer. These two types of transfer learning are also widely used in multitasking DRL algorithms.

Regarding SGS for detecting of pulmonary pathogen infection, multiple agents and multitask are promising. Therefore, the deep reinforcement learning based on multitask transfer learning is a feasible direction.

*(3) Deep Reinforcement Learning Based on Memory and Reasoning*. The traditional visual-perception-based DRL method is far worse than human beings in solving higher-level cognition-inspired tasks. That is to say, in solving some high-level DRL tasks, the agent not only needs strong perceptual ability, but also needs certain memory and reasoning ability to learn effective decision-making. Therefore, the ability to give active learning and reasoning to existing DRL models is very important.

In recent years, the research on neural network model of external storage has made substantial progress. Graves et al. [28] proposed a neural structure called Neural Turing Machines (NTM), which updates the parameters of memory structures by random gradient descent while reading and writing data to optimize the content of memory. By adding NTM, the neural network model has the ability to complete some simple tasks such as copying, inversion, addition, and subtraction, which shows that the deep neural network model has preliminary memory and reasoning ability. After that, Sukhbaatar et al. [29] proposed a memory network model based on NTM for question-and-answer system and language modelling tasks, which further improved the long-term memory ability of the network. Therefore, adding these external memory modules to the existing DRL model can give the network a certain high-level ability of long-term memory, active cognition, and reasoning. In addition, the development of cognitive neuroscience in recent years has also promoted the development of artificial intelligence. Researchers are simulating the auxiliary learning system of the human brain to construct an agent that can independently remember, learn, and make decisions.

## 3. Current Status of Pathogenic Diagnosis of Pulmonary Infectious Diseases

In recent years, as a result of the emergence of severe acute respiratory syndrome (SARS) in new pathogens, a variety of viruses, fungi, and resistant bacteria have emerged, and infectious diseases have once again received attention. However, there are many kinds of pathogens of infectious diseases [30–32]. Traditional immunological tests and cultivation of pathogenic microorganisms are limited; they are not efficient and timely to provide a reliable diagnosis basis for the clinic, especially the complex lung infection. How to judge the pathogens? Difficulties have made many patients with pulmonary infectious diseases fail to receive timely and effective treatment and even death. Pulmonary infections are mainly pneumonia and bronchiolitis and can also be manifested as lung abscesses and granulomas. Pulmonary infections are mainly collected from sputum and alveolar lavage fluid. Due to the special feeding environment of the lungs, routine detection of effective pathogen information in serum is limited. However, patients with severe pneumonia require mechanical ventilation, and lung tissue is difficult to obtain. The small amount of specimens limits the detection of pathogens of infectious lung diseases.

## 4. Application of SGS in Detection of Pulmonary Pathogen Infection

At present, pathogenic diagnostic methods based on microbial culture are still the main means for diagnosing the pathogenic diagnosis of pulmonary infectious diseases, but they are also largely influenced by culture conditions and antibiotic use, and the culture positive rate is low. For lung infections, second-generation sequencing technology can be used for the detection of a variety of pathogens, such as bacteria, fungi, viruses, mycoplasma, etc. Also, it can also be used for the detection of a variety of respiratory specimens, such as sputum, throat swab, alveolar lavage fluid and blood, and other specimens. The second-generation sequencing technology has a higher detection rate than the traditional culture method [33, 34]. Compared with the traditional single-plex PCR method, it not only reduces the sample nucleic acid requirement and expands the detection range, but also has better specificity and sensitivity [11, 12]. Detection of viral pathogens mainly includes virus antigen detection, nucleic acid detection, and virus isolation and culture. However, the traditional virus pathogen detection has a low positive rate; thus, it is difficult to promote and apply in clinical practice. Second-generation sequencing technology is superior to traditional virus detection techniques in terms of sensitivity and accuracy. It theoretically reveals all microbial information in the sample, which can detect more virus types and its positive rate is higher [35]. Detectable viruses include well-known upper respiratory tract viruses and lower respiratory tract viruses such as HSV and CMV in immunosuppressed hosts. With the increasing range of pathogens based on second-generation sequencing technology, it is found that the proportion of viruses in respiratory infections is much higher than previously thought. The applications of SGS technology to detect in-hospital-acquired viral pneumonia in real time and rapid detection have shown that SGS technology can learn evidence faster than traditional methods, so that timely measures are taken to control outbreaks of nosocomial infections.

Pulmonary fungal infections are characterized by high lethality and difficulty in diagnosis and treatment. In recent years, with the abuse of antibiotics, more and more resistant bacterias and fungi have emerged. Pulmonary fungal infection has become the leading cause of death in ventilator-associated pneumonia, especially in patients with immunosuppression. Among patients with drugs, the incidence of fungal infections is also increasing [36]. Culture has long dominated the diagnosis of fungal infections, but traditional methods have inherent deficiencies in identifying mixed infections and analyzing the flora structure and dynamics of the flora and many fungi or undiscovered new ones. It is difficult or even impossible to cultivate the strain. These kinds of problems all suggest that we urgently need a new method to assist clinical diagnosis more accurately and quickly. Different from the bacterial DNA extraction method, the second-generation technology in the fungal flora structure spectrum method mainly through the amplification, sequencing, and analysis of the fungus ITS1 (internal transcribed spacer) and ITS2 gene fragments, using ITS1/ITS2 gene sequencing technology, generally, 50 to 60 genera were detected, and the sequence of fungal ITS gene obtained by sequencing can be matched by the existing gene database. However, for the most common clinical genera of *Candida* and *Aspergillus*, ITS gene sequencing can well identify their ITS gene sequences, and some pathogen strains can be distinguished at the species level. Fungi not only cause lung dysfunction, but also because of the long treatment time, affect the prognosis of the disease, and the second-generation sequencing technology helps to understand the whole picture of airway microbes from the overall structure of the community and in complex lung infections, especially AIDS, etc. The diagnosis of *Pneumocystis* is important in immunodeficient patients [37].

Mycobacterium tuberculosis also occupies an important position in the pathogens of lung infection. At present, the commonly used PCR method for collection of mycobacterium tuberculosis has low sensitivity and low positive rate. The T-SPOT detection method is also applied clinically, but the specificity is low, and the false positive rate is high. However, the second-generation sequencing technology, the pathogen detection, epidemiology, and typing of mycobacterium tuberculosis have made a leap forward. The second-generation sequencing technology can be applied not only to conventional sputum, alveolar lavage fluid and blood, but also to the detection of pleural effusion and pericardial effusion, which greatly improves the accuracy and sensitivity of detection, especially improving the diagnosis of tuberculous pleural effusion. At the same time, SGS can classify the detected *M. tuberculosis*, greatly improve the typing efficiency, and also determine the variation and propagation source among the strains in the transmission chain.

It is well known that the clinical microbiology laboratory's real-time PCR technology for screening and confirming suspected viruses must be based on known pathogen gene sequences, but not for unknown viral pathogens. The second-generation sequencing technology not only discovers known pathogens, but also discovers completely unknown pathogens [38, 39]. About 70% of patients with infectious diseases cannot determine pathogen information due to traditional detection methods and cannot be treated in a timely and effective manner, thus worsening the condition. Therefore, rapid and accurate pathogen detection methods are of great significance for effective diagnosis and timely control of infectious diseases. There are many types of modern molecular typing techniques, and the most commonly used molecular techniques in the traceability and monitoring of infectious diseases are multisequence typing (MLST), pulsed-field gel electrophoresis (PFGE), and multisite tandem repeats. Sequence analysis (MLVA), etc., and the emerging high-resolution WGS technology guarantee the accurate traceability and monitoring of infectious diseases and can also complement each other with multiple molecular technologies to improve the accuracy of detection. WGS technology can track the prevalence of pathogens and more accurately identify possible sources of pathogens [40, 41]. And with the continuous development of this technology, the current traceability and monitoring capabilities for unknown pathogens are becoming more and more prominent. The pathogens of severe pulmonary infection are usually unclear. Currently, the clinical use of antigen/antibody immunology methods and traditional microbial culture techniques is used for diagnosis. However, these methods have problems of long culture period and low culture positive rate. The second-generation sequencing technology is a novel DNA/RNA sequencing method based on the detection of nucleic acid molecules, which has high sensitivity and short time-consuming, and does not depend on traditional pathogenic culture. The application of antibiotics in the early stage has little effect on the detection results. Accurate and rapid identification of microbial pathogens in patients with lung infections may result in targeted antibacterial therapies, with fewer side effects and lower costs. In particular, patients with tracheal intubation are measured by sputum extraction from the lower respiratory tract of the bronchus, which can more accurately provide pathogenic bacteria analysis of ventilator-associated pneumonia, further guiding clinical treatment and prognosis. With the development of second-generation sequencing technology, processing and sequencing time will be further reduced. The SGS method will eventually provide clinicians with rapid, accurate, independent culture-based identification of bacterial, fungal, and viral pathogens and their antimicrobial sensitivity characteristics [42].

Although the blood and alveolar lavage SGS tests have revolutionized the pathogens of complex lung infections, there are still many problems with the detection and interpretation of SGS. Different from the application of SGS detection in hereditary diseases, the complex composition of infectious disease specimens and the low level of nucleic acid of pathogenic microorganisms all restrict the detection of SGS in pathogenic microorganisms of lung infection. The first problem is sensitivity. Although SGS detection shows great advantages over traditional methods in diagnosing rare and rare pathogens, the sensitivity of common pathogens such as *Cryptococcus* is not stronger than traditional methods. Even if these pathogens detected by traditional methods, SGS may not detect or detect only a very small number of specific fragments, which affects the results. For example, for the metagenomics detection of *M. tuberculosis*, the current optimal solution is still to perform SGS sequencing on the basis of MGIT960 liquid culture. The reason may be that the *Mycobacterium tuberculosis* are intracellular bacteria, and the current detection method is mainly to detect the intracellular infection with *M. tuberculosis* by using the sputum supernatant; and *M. tuberculosis* due to nucleic acid of GC is relatively high (60% higher), and the melting method of most bacteria cannot fully dissociate the nucleic acid strand of *M. tuberculosis*. Therefore, under the condition of specimen processing suitable for most pathogenic microorganisms, the detection of tuberculosis cannot be effectively achieved. At present, direct SGS testing of clinical specimens to diagnose *M. tuberculosis* infection is very difficult. As an alternative, it is often the case that the clinical specimens are cultured for tuberculosis and then SGS is detected to improve the positive rate of clinical specimen detection, which increases the financial burden of the patient.

## 5. Discussion

Although SGS is a high-throughput test and can theoretically detect almost all pathogenic microorganisms, it does not completely replace all clinical pathogen detection methods. This requires us to continue to optimize the detection method of SGS on the one hand, so that it can cover the range of pathogens as much as possible and improve the detection rate. On the other hand, we must understand that SGS technology has limitations and cannot be completely dependent on SGS detection [43]. At the same time, traditional methods cannot be ignored, and other effective pathogen detection methods should be retained or explored as supplements or verifications. For patients with partial lung abscess or granuloma, because the disease is confined to the lungs, the amount of pathogens released into the blood and alveolar lavage fluid is limited, so for these diseases, pathogens can only be detected by SGS detection of tissue samples. Deep reinforcement learning can fully learn the potential results by simulating human brain learning and decision making based on the given complex data. Thus, it is a good choice to integrate DRL with SGS for clinical pathogen detection.

Poor specificity is another major problem that restricts the clinical application of SGS. Nonpathogenic, unrelated pathogens, and ambiguous pathogens are often seen in SGS reports. The lungs are an open environment, connected to the outside world, there are airway and oral colonization bacteria, many fragments of different species can be detected in the sputum and alveolar lavage fluid, and many unexplained samples can often be detected in the specimen. Part of the reason may be the contamination of specimens and reagents, such as environmental microbes (such as plants, plant viruses, etc.), which are difficult to discriminate. For these contaminations, it is necessary to eliminate the comparison between the laboratory quality control and the data between the specimens. DRL can combine all the related data to learn a favorable result through training and decision making; thus, it may effectively solve the poor specificity of the clinical application of SGS.

In summary, by combining the deep reinforcement learning techniques with SGS, it can eliminate the valueless results and analyze and evaluate the meaningful results of SGS. The core of SGS testing for the diagnosis of lung infections is the identification of responsible pathogens. Since SGS testing often yields a large number of backgrounds or unrelated microbial fragments, it is critical to find or identify responsible pathogens. It is first necessary to establish a knowledge database of common microorganisms for lung infections: a database of background microorganisms common to each laboratory and testing unit in SGS testing and record the number of common fragments detected. If a suspected pathogen fragment that is not in the range of common background bacteria appears in the SGS test of clinical specimens, or the number of fragments of a certain microorganism is significantly higher than the data in the background microbial database, it is included in the suspected responsible pathogen, and further methods are used for authenticating. In the alveolar lavage fluid, possible responsible bacteria, fungi, or virus fragments were detected by SGS, and the proportion of the total fragments was often extremely low, even only a few fragments, which is difficult to be diagnosed. SGS testing is often only useful in the diagnosis of pulmonary systemic infections, and when pathogens for nonclinical routine screening are detected, further use of classical pathogen detection methods is needed for diagnosis. This requires the development of a well-developed pathogen verification test system, especially for pathogenic microorganisms that are not easily detected by some common methods. Because the current SGS testing cost is still high, it cannot be widely used in the clinic, and it also affects its timeliness. SGS is also only a pathogen detection method, and it has just been applied in clinical practice. Therefore, there are also blind spots and misunderstandings of its monitoring. The excessive expectation and interpretation of SGS detection results can not only push up the cost of clinical testing, but also make it effective. The examination could not be carried out smoothly, which also led to misdiagnosis and missed diagnosis.

However, because the optimal processing conditions and bioinformatics analysis required for sequencing of different specimens and pathogenic microorganisms are different, it is currently not possible to adapt an SGS detection procedure to all infectious pathogens. Moreover, the difference in the location and method of the specimen will also affect the test results. Therefore, using the deep reinforcement learning, based on the patient's medical history and clinical examination, the possible pathogens are presumed, the specimens are preprocessed and then sequenced, and even different strategies are adopted for the biosignal analysis of the sequencing results. Taking into account various unknown pathogens, the detection rate of specific pathogens and the interpretation of the results are improved. The deep reinforcement learning is a diagnostic basis to reduce errors. It sets certain standards and procedures to determine whether the detected pathogen is a responsible pathogen, and designing targeted evaluation sequencing methods based on different types of pathogens to improve the effectiveness of SGS. Therefore, the deep reinforcement learning combined with knowledge graph is a promising direction for SGS in the application of pulmonary infectious diseases.

## 6. Conclusion

In this paper, we explore deep reinforcement learning for computer-aided diagnosis and treatment of complex pulmonary infectious diseases. We first present several representative deep reinforcement learning models for the diagnosis and treatment of potential lung infections. Moreover, we discuss the applications of the deep reinforcement learning model in the diagnosis of second-generation genetic testing for pulmonary infection and summarize the current status of pathogenic diagnosis of pulmonary infectious diseases and their main characteristics. After that, we analyze the results of second-generation genetic testing and the main features of each type in common lung infections. Finally, we point out the open challenges and possible future research directions for intensive studies of second-generation genetic testing integrated with deep reinforcement learning in lung infections, which may help the related researchers and medical workers.

## Data Availability

The data used to support the findings of this study are currently under embargo, while the research findings are commercialized. Requests for data will be considered by the corresponding author.
